# Leukocyte count and incidence of subarachnoid haemorrhage: a prospective cohort study

**DOI:** 10.1186/1471-2377-14-71

**Published:** 2014-04-03

**Authors:** Martin Söderholm, Elisabet Zia, Bo Hedblad, Gunnar Engström

**Affiliations:** 1 Cardiovascular Epidemiology Research Group, Department of Clinical Sciences Malmö, Lund University, CRC building 60 floor 13, Jan Waldenströms gata 35, 20502 Malmö, Sweden

**Keywords:** Subarachnoid haemorrhage, Inflammation, Leukocytes, Epidemiology

## Abstract

**Background:**

Subarachnoid haemorrhage (SAH) is a devastating disease, in the majority of cases caused by a rupture of an arterial intracranial aneurysm. The effect of systemic low-grade inflammation on incidence of SAH is not known. The purpose of this study was to evaluate the relationship between leukocyte count, a marker of systemic inflammation, and incidence of SAH in a large cohort study.

**Methods:**

Leukocyte count and other cardiovascular risk factors were measured in 19,794 individuals (17,083 men and 2,711 women, mean age 44 years) participating in a health screening program between 1974 and 1981. Incidence of SAH in relation to baseline leukocyte concentration was studied during a mean follow-up of 27 years in participants free from previous stroke.

**Results:**

Ninety-five participants had a SAH, corresponding to an incidence of 22 per 100,000 in women and 17 per 100,000 in men. The hazard ratio for SAH per one standard deviation (2.01 × 10^9^ cells/L) increase of leukocyte concentration was 1.26 (95% CI 1.05-1.53, p = 0.014) after adjustment for several potential confounding factors including smoking. In sensitivity analysis, there was a significant association in smokers but not in non-smokers.

**Conclusions:**

High leukocyte count at baseline was associated with increased incidence of SAH, although this relationship might be restricted to smokers. The results support the view that low-grade systemic inflammation could be involved in the pathogenesis of SAH, or constitute an early risk marker for the disease.

## Background

Subarachnoid haemorrhage (SAH) constitutes approximately 5% all strokes [1]. SAH is associated with a high mortality and morbidity [2] and is a disease of high clinical importance. In the majority of cases (approximately 85%) SAH is caused by a rupture of an arterial intracranial aneurysm [3,4]. Important risk factors for SAH are female sex, family history, hypertension, smoking and excessive alcohol consumption [4,5].

The pathogenic mechanisms leading to aneurysm formation and rupture are, however, not fully understood. Infiltration of inflammatory cells and increased expression of inflammatory mediators have been observed in the aneurysm wall in histological studies, and are more pronounced in ruptured than unruptured aneurysms. These inflammatory changes might occur prior to aneurysm formation and rupture, or be a reaction to the degeneration of the aneurysm wall [6].

Systemic low-grade inflammation, measured as levels of leukocytes, high sensitive C-reactive protein (hsCRP) or fibrinogen, is involved in development of atherosclerosis and associated with incidence of ischemic stroke and coronary heart disease in prospective studies [7-12]. There is also an immuno-inflammatory activation after acute ischemic stroke onset, contributing to the development of acute brain ischemia [13,14]. Plasma levels of several immuno-inflammatory markers are associated with a diagnosis of ischemic stroke, [14] although the plasma markers could differ between subtypes of ischemic stroke [13].

It is not known whether systemic low-grade inflammation is associated with the incidence of SAH, and only two prospective studies have addressed this question. In one study incidence of SAH was evaluated in relation to hsCRP [15] and in another study in relation to fibrinogen [10]. No significant associations were found in any of the studies, possibly due to the low numbers of SAH events. Leukocyte concentration in relation to incidence of SAH has, to our best knowledge, not earlier been studied. In this study we investigated the association between leukocyte count and incident SAH in a large prospective cohort study from an urban population.

## Methods

### Study population

Between 1974 and 1992, a screening program with the aim to identify individuals at high risk for cardiovascular disease, the Malmö preventive project, was conducted in Malmö, Sweden [16]. Complete birth cohorts, born between 1921 and 1949, were invited to a health examination including a physical examination, a panel of laboratory tests and a self-administered questionnaire. A total of 22,444 men were examined from 1974 to 1984 and 10,902 women were examined between 1977 and 1992. The Health Service Authority of Malmö approved the screening program, and all participants gave informed consent. Linkage with the national hospital discharge register and the causes of death register was approved by the ethics committee at Lund University.

In the Malmö preventive project, leukocyte count was measured in participants invited between 1974 and 1981. During this period, men born in 1921, 1926–1940, 1942, 1944, 1946 and 1949 were invited, and women born in 1926, 1931, 1938, and 1949. A total of 19,930 subjects, 2,732 women and 17,198 men, participated. Participation rate was 71% both for men and women.

Subjects with a history of stroke (n = 10), and those with missing information about systolic blood pressure (n = 14), total cholesterol (n = 28) and diabetes (n = 81) were excluded. We also excluded seven individuals with leukocyte counts >20.0 × 10^9^ cells/L as extreme outliers from the population distribution, since they may have had a subclinical hematologic disease or laboratory error at baseline [12]. A total of 19,794 participants (2,711 women and 17,083 men) with a mean age ± SD of 44 ± 6 years remained for the analysis. Mean age in women was 42 ± 9 years (range 28–54) and in men 44 ± 5 years (range 27–61).

### Baseline examinations

Blood pressure (mm Hg) was measured twice in the right arm after 10 minutes of rest. The average of two measurements was used. A sphygmomanometer and a rubber cuff of appropriate size were used. Height (cm) and weight (kg) were measured under standardized conditions and body mass index was calculated as weight/height^2^ (kg/m^2^). Current smoking habits, alcohol consumption and use of antihypertensive medication were evaluated in a questionnaire. Subjects were categorized in terms of smoking as never smokers, former smokers (i.e. non-smokers who previously had been smoking daily for at least 6 months) and current smokers (i.e. daily smoking).

Alcohol abuse was assessed by means of the modified shortened version of the Michigan Alcoholism Screening Test [17]. Subjects with more than two (of nine) affirmative answers were considered to have high alcohol consumption. Information about previous myocardial infarction (MI) before screening was based on the questionnaire and data from the Swedish hospital discharge register. Subjects who confirmed a doctor’s diagnosis of angina pectoris or who used nitrates were considered to have angina pectoris (AP). In the present study prevalent MI and AP was combined into one variable; history of MI or AP. Physical inactivity in spare time was assessed using the question: ‘Are you mostly engaged in sedentary activities in spare time, for example, watching TV, reading, going to the movies?’.

Blood samples were taken after an overnight fast. Leukocyte count was determined with automatic counters in accordance with standard methods at the hospital laboratory. Serum cholesterol and blood glucose were analysed with standard methods. Diabetes was defined as fasting whole blood glucose > 6.1 mmol/L or self-reported treatment for diabetes. Forced expiratory volume in one second (FEV1) was measured in a subsample (n = 18,607, 94%) of the present cohort, using spirometry. The methods for the lung function assessment and standardization of FEV1 to percentages of the individuals predicted value have been described elsewhere [18].

### Retrieval of cases

All subjects were followed from the baseline examination until first SAH, death, emigration from Sweden or December 31, 2008, whichever came first. SAH cases were identified with the Malmö stroke register, the Swedish hospital discharge register or the causes of death register (ICD 8 and 9 code 430, and ICD 10 code I60), as previously described [18]. These registers provide complete coverage of hospital discharges and deaths in Sweden. All diagnoses are made by a physician at hospital discharge or death of the patient, and contra-signed by board-certified specialists [19].

### Statistical analysis

Leukocyte count was categorized into sex-specific quartiles with equal proportions of women in each quartile, in order to adjust for the relationship between sex and SAH. One-way analysis of variance (for continuous variables) and logistic regression (for categorical variables) were used for cross-sectional analyses of the risk factor distributions at baseline across quartiles of leukocytes. Cox proportional hazards regression was used to calculate risk factor-adjusted hazard ratios (HR) and 95% confidence intervals (CI). Leukocyte concentration was evaluated as a continuous variable (per 1-SD increase) and in sex-specific quartiles. Unadjusted results and results from a multivariable model adjusted for the categorical variables sex, smoking status, antihypertensive medication, high alcohol consumption, diabetes and physical inactivity, and the continuous age, systolic blood pressure, body mass index, cholesterol, are presented. To explore whether FEV1 or a history of MI/AP, explained the relationship between leukocytes and SAH, these covariates were entered separately to the multivariable model. Covariates were selected because they have shown associations with SAH [5] and systemic inflammation [20], respectively, and thus are potential confounders of the association between SAH and leukocytes. The fit of the proportional hazards model was checked visually by plotting the incidence rates over time, and by plotting Schoenfeld residuals as a function of time and statistically testing for a non-zero slope. Possible interactions between leukocyte count and each covariate, respectively, were checked by interaction terms added to the full model. The Cox regression analyses were also performed separately in current, former and never smokers, and in women and men. Harrell’s C statistics was used to estimate the ability of the risk factor adjusted Cox model to predict SAH.

## Results

### Baseline characteristics

Baseline characteristics by quartiles of leukocytes are described in Table 1. Leukocyte count was significantly associated with systolic blood pressure, current smoking, high alcohol intake, body mass index, total cholesterol, diabetes and physical inactivity.

**Table 1:** **Baseline characteristics by sex**-**specific quartiles of leukocyte count**

	**Q1**	**Q2**	**Q3**	**Q4**	**p trend**	**All**
n	5110	4864	4794	5026		19,794
Leukocytes, 10^9^ cells/L	3.93 ± 0.5	5.17 ± 0.3	6.33 ± 0.4	8.74 ± 1.6		6.04 ± 2.01
Leukocytes, 10^9^ cells/L, range	2.0-4.6	4.6-5.7	5.6-7.0	7.1-19.2		2.0-19.2
Women, %	13.4	13.1	15.1	13.2		13.7
Age, years	43.6 ± 6.1	43.8 ± 6.0	43.7 ± 6.1	43.9 ± 5.9	0.055	43.7 ± 6.0
Systolic blood pressure, mmHg	126 ± 14	127 ± 15	127 ± 16	127 ± 16	0.028	126 ± 15
Smoking status, %						
Never smokers	50.2	38.2	27.5	13.2	< 0.001	32.3
Former smokers	24.6	22.4	16.4	8.4	< 0.001	18.0
Current smokers	25.2	39.4	56.1	78.4	< 0.001	49.7
High alcohol consumption, %	12.1	13.3	14.5	17.6	< 0.001	14.4
Antihypertensive medication, %	3.4	3.6	4.1	4.0	0.057	3.8
Body mass index, kg/m^2^	24.2 ± 3.1	24.6 ± 3.4	24.7 ± 3.6	24.4 ± 3.5	0.002	24.5 ± 3.4
Cholesterol, mmol/L	5.51 ± 1.0	5.58 ± 1.0	5.62 ± 1.0	5.76 ± 1.1	< 0.001	5.61 ± 1.1
Diabetes, %	2.1	2.2	2.6	2.9	0.005	2.5
Physical inactivity, %	50.9	54.8	56.7	58.9	< 0.001	55.3
History of MI or AP, %	1.8	1.5	2.0	2.1	0.093	1.8
FEV1, % of predicted	97.5 ± 17.6	95.6 ± 17.4	93.4 ± 17.4	91.1 ± 17.5	< 0.001	94.4 ± 17.7

Values are mean ± SD or percentages.

Q, quartile; MI, myocardial infarction; AP, angina pectoris; FEV1, forced expiratory volume in 1 s (n = 18,607).

### Incidence of SAH

During the mean follow-up of 26.8 ± 6.9 years (530,562 person years), ninety-five subjects (78 men and 17 women) had a SAH, corresponding to a crude incidence of 17.9 per 100,000 person years (17.2 in men and 22.1 in women). Mean age at SAH was 58.1 ± 11 years and mean time from screening to SAH was 15.1 ± 8.4 years.

Higher leukocyte concentration was significantly associated with higher incidence of SAH. Per 1 SD (2.01 × 10^9^ cells/L) increase of leukocytes, the crude HR (95% CI) for SAH was 1.30 (1.09-1.54), p = 0.003, and the HR after adjustment in the multivariable model 1.26 (1.05-1.53), p = 0.014 (Table 2). After additionally adjusting for FEV1 (in % of the predicted values) HR was 1.25 (1.04-1.52), p = 0.020, per SD increase of leukocytes. Further adjusting for history of MI/AP did not substantially change the result.

**Table 2:** **Hazard ratios for subarachnoid haemorrhage per 1 standard deviation increase of leukocytes and in relation to sex**-**specific quartiles of leukocytes**, **for all participants and stratified by sex and smoking status**

	**Q1**	**Q2**	**Q3**	**Q4**	**Per 1-SD increase**	**p value**
*All*						
n	5110	4864	4794	5026	19794	
SAH, n	15	23	27	30	95	
HR (95% CI)	1	1.65 (0.86-3.16)	2.00 (1.06-3.76)	2.22 (1.19-4.12)	1.30 (1.09-1.54)	0.003
HR*	1	1.63 (0.85-3.14)	1.92 (1.00-3.66)	2.05 (1.06-3.96)	1.26 (1.05-1.53)	0.014
*Men*						
n	4423	4227	4072	4361	17083	
SAH, n	11	20	22	25	78	
HR	1	1.95 (0.93-4.07)	2.27 (1.10-4.69)	2.53 (1.25-5.15)	1.28 (1.05-1.55)	0.013
HR*	1	1.99 (0.95-4.18)	2.35 (1.12-4.94)	2.63 (1.23-5.62)	1.28 (1.04-1.59)	0.021
*Women*						
n	687	637	722	665	2711	
SAH, n	4	3	5	5	17	
HR	1	0.81 (0.18-3.64)	1.21 (0.32-4.51)	1.32 (0.36-4.93)	1.37 (0.95-1.97)	0.095
*Never smokers*						
n	2563	1858	1317	665	6403	
SAH, n	5	10	8	2	25	
HR	1	2.80 (0.96-8.18)	3.18 (1.04-9.72)	1.60 (0.31-8.24)	1.31 (0.82-2.11)	0.257
*Former smokers*						
n	1249	1085	783	420	3555	
SAH, n	8	5	3	2	18	
HR	1	0.74 (0.24-2.26)	0.62 (0.16-2.34)	0.81 (0.17-3.80)	0.93 (0.49-1.78)	0.824
*Current smokers*						
n	1288	1908	2635	3913	9836	
SAH, n	2	8	16	26	52	
HR	1	2.72 (0.58-12.8)	3.88 (0.89-16.9)	4.47 (1.06-18.8)	1.33 (1.07-1.65)	0.010
HR*	1	2.73 (0.58-12.8)	3.91 (0.90-17.0)	4.51 (1.07-19.0)	1.32 (1.06-1.63)	0.011

*Adjusted for age, sex, systolic blood pressure, antihypertensive medication, smoking status, high alcohol consumption, body mass index, diabetes, total cholesterol and physical inactivity. This model was not applied in all subgroups, due to limited number of events.

1 SD; 2.01 × 10^9^ cells/L. Q; quartile. SAH; subarachnoid haemorrhage. HR; hazard ratio.

To explore if the relationship between leukocytes and SAH changed with time, the follow-up was divided at the median follow-up time for those with SAH (14.6 years) and separate models was fitted for the two intervals. The HR was 1.33 (1.03-1.67) and 1.28 (1.00-1.64), respectively, in the first and second time period (p for interaction with time 0.9).

There were no statistically significant interactions between leukocytes and other variables in the analysis. In subgroup-analysis, the association between SAH incidence and leukocyte concentration (per 1 SD) remained significant in men (p = 0.021) and in smokers (p = 0.011) after full adjustment. The full multivariate model could not be applied in all subgroups due to the limited number of events (Table 2).

**Figure 1:**
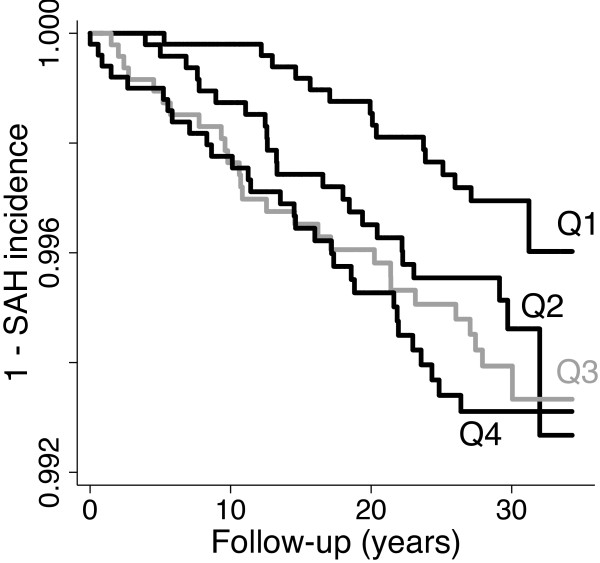
Incidence of subarachnoid haemorrhage by sex-specific quartiles of leukocytes.

The association between leukocyte count and incident SAH was significant in a dose–response manner when leukocyte concentration was analysed in sex-specific quartiles (unadjusted p for trend = 0.010), Table 2 and Figure 1.

Adding leukocyte concentration (continuous/per 1 SD) to the other variables in the full multivariable model increased the SAH predictive ability of the model (c statistics 0.6248 vs. 0.6439).

## Discussion

In the present prospective cohort study, systemic inflammation measured by the leukocyte count was associated with incidence of SAH in a representative sample of healthy middle-aged subjects from the general population. The association persisted during a long follow-up time, also after adjustment for established SAH risk factors [5] including smoking. However, the association between SAH and leukocytes might be limited to smokers.

To our best knowledge, no study has previously evaluated leukocyte count and incidence of SAH. A relationship between fibrinogen and incidence of haemorrhagic stroke has been shown in a meta-analysis but no distinction of SAH and ICH was made [9]. Two prospective studies have examined the effect of markers of systemic inflammation, hsCRP [15] and fibrinogen, [10] in relation to SAH incidence. No significant associations were found in any of the studies. However, because the studies included only 29 and 33 SAH cases, respectively, they could be underpowered. Some published studies could provide indirect support to the hypothesis of systemic inflammation in development of SAH. For example, polymorphisms of the interleukin 6 gene, a stimulator of inflammatory plasma proteins, have shown association with intracranial aneurysm prevalence [21] and an increased risk of SAH has been found in patients with systemic lupus erythematosus [22]. It needs to be further studied whether other systemic markers of inflammation (e.g. CRP or interleukin 6) are associated with SAH and if their ability to predict SAH is higher in comparison with leukocytes.

Smoking is one of the most important risk factors for SAH [5]. The relationship between leukocytes and SAH was significant after adjustment for smoking, but in subgroups analysis, a significant association was only observed in smokers but not in non-smokers. However, there was no significant interaction between leukocytes and smoking. The absence of an association in non-smokers might be due to low statistical power leading to the association being undetected. The observation of a similar point estimate for the HR in never smokers and smokers might support this view, although this is not true for former smokers. Alternatively, the relationship between leukocytes and SAH could be an effect of smoking, i.e. inflammation could be a mediator of the increased SAH risk in smokers. If so, leukocytes could be a marker of increased susceptibility to the deleterious effects of smoking. As previously suggested, this could work through neutrophils secreting proteolytic enzymes in response to cigarette smoke, promoting degradation of elastin and collagen and thus formation of aneurysms [23]. Although we do not have data on neutrophil level, this study then adds to the knowledge by showing that levels of leukocytes, not just their enzymes, are elevated in smokers developing SAH many years before the event. Larger studies will be needed to determine if leukocyte count is a risk factor for SAH also in non-smokers.

It is not known if SAH can be caused by atherosclerosis, but atherosclerosis can be present in cerebral aneurysms [24]. Because atherosclerotic disease is associated with raised leukocytes, this might contribute to the relationship between leukocytes and SAH. However, in the present study there were no significant interactions between leukocytes and other cardiovascular risk factors on incidence of SAH, and adjusting for a history of MI or AP did not change the results. It should be acknowledged, though, that interaction analyses require relatively high number of events, and the statistical power might be too low for these analyses.

The main strengths of this study include the prospective population-based design, a high participation rate of middle-aged, healthy subjects from an urban population, a long follow-up time, and a relatively high number of SAH cases. Further, using hospital-based registers for case retrieval is appropriate because all non-fatal SAH patients should be admitted to hospital. SAH cases dying unexpectedly outside of hospital were identified with the causes of death registry, and for these cases the diagnosis was based on autopsy in 10 cases out of 11. In the local stroke register all diagnoses are verified by review of the patient records. SAH diagnoses identified with the national hospital discharge registry were reviewed with respect to which clinic the patient was treated in and what surgical procedure that was undertaken to treat SAH.

Some limitations need to be considered. Despite the relatively high number of SAH events in the present cohort, events are too few to perform analysis of all subgroups. Second, elevated leukocyte count is a non-specific sign of inflammation and there could be many underlying causes. Some individuals might have had acute infections causing elevation of leukocytes at baseline. However, misclassification of some individuals with high leukocytes due to acute infection would weaken the association between leukocytes and SAH. Even though the cohort consists of individuals from the general population, it is also possible that a few participants had a systemic inflammatory disease, e.g. SLE or rheumatoid arthritis. Even though these disorders are uncommon in men from this age group, comorbid conditions could still contribute to the association between leukocytes and SAH.

There are also several drugs, e.g. non-steroid anti-inflammatory drugs, statins, corticosteroids and blood pressure drugs, which could theoretically influence the level of leukocytes and perhaps also the risk of SAH. Blood pressure medication was slightly more common in those with higher leukocytes, and adjustment for this did not change the results substantially. Statins were not on the market when the baseline examinations were performed, and aspirin was not used for prevention of cardiovascular disease at that time. We had no information about the other mentioned drugs. There is no obvious reason to believe that medication of any kind could explain the present results. Further studies are needed to explore whether anti-inflammatory drugs also could reduce the risk of SAH.

Third, we included all non-traumatic SAHs. Approximately 85-90% of non-traumatic SAHs are aneurysmal, so the results do not represent only aneurysmal SAH. Further, leukocytes were only measured at one time-point, and we do not know to what extent leukocyte concentrations changed during follow-up. It has been shown that leukocyte concentrations are rather stable over time [25] and substantially influenced by genetic factors [26]. The present results also show that the association between leukocytes and SAH was essentially unchanged with time, which is unlikely if there are great intra-individual variations. Misclassification of the exposure due to individual biological variation over time will be non-differential and dilute the association between leukocytes and SAH [27]. This is also supported by previous studies taking into account the variation over time when assessing the relationship between leukocytes and cardiovascular disease [28].

## Conclusions

In conclusion, high leukocyte count at baseline was associated with increased incidence of SAH, although this might be restricted to smokers. The results support the view that inflammation might precede the development of SAH and mediate the effect of smoking on SAH.

## Competing interests

The authors declare that they have no competing interests.

## Authors’ contributions

MS and GE contributed to the concept and design, analysed and interpreted data, drafted and revised the manuscript. EZ and BH contributed to the concept and design, and revised critically the manuscript for important intellectual content. All authors approved the final version.

## Pre-publication history

The pre-publication history for this paper can be accessed here:

http://www.biomedcentral.com/1471-2377/14/71/prepub
